# Genetic mapping of Ascochyta blight resistance in an ILL6002 × Indianhead lentil mapping population

**DOI:** 10.1002/tpg2.70097

**Published:** 2025-08-30

**Authors:** Em L. Thackwray, Bernadette M. Henares, Christina R. Grime, Bethany L. Clark, Robert C. Lee, Lars G. Kamphuis

**Affiliations:** ^1^ Centre for Crop and Disease Management, School of Molecular and Life Sciences Curtin University Bentley Western Australia Australia

## Abstract

Ascochyta blight of lentil (*Lens culinaris* Medik.) is a fungal disease caused by *Ascochyta lentis*. This study was carried out to identify the location of quantitative trait loci (QTL) associated with resistance from the accession Indianhead, and how these vary between the recently identified pathotypes of *A. lentis*. We performed QTL mapping using F_6_ recombinant inbred lines derived from a cross between the resistant cultivar Indianhead and susceptible accession ILL6002, following evaluation in seedling assays and the field. Phenotyping identified nine QTL across the four different isolates. A major QTL effective against Pathotype 1 isolates was identified on chromosome 2 in both the seedling and field phenotyping, explaining 60.5% and 12.6% of the resistance phenotype, respectively. Additional QTL for resistance associated with Pathotype 1 isolates were identified on chromosomes 3, 5, and 7, explaining between 8.5% and 13.1% of the phenotype. In contrast, QTL associated with resistance to Pathotype 2 isolates were identified on chromosomes 1, 2, 3, and 7, in locations distinct from those associated with Pathotype 1 resistance. These loci explained between 8.8% and 29.6% of the phenotypic variation. Additionally, evaluation of a natural powdery mildew infection revealed a major QTL on chromosome 3, explaining 25% of the resistance phenotype. The markers flanking the loci identified herein will allow for lentil breeding programs to trace the associated resistance in their breeding program pedigree.

## Core Ideas

- Generated a lentil recombinant inbred line population derived from cultivar Indianhead and accession ILL6002 to evaluate for Ascochyta blight resistance.
- Eight distinct loci effective against Pathotype 1 and Pathotype 2 were identified from seedling and field assays for Ascochyta blight resistance.
- A natural powdery mildew infection was scored, and quantitative trait locus (QTL) analyses identified a major QTL on chromosome 3.

## Plain Language Summary

Lentil is a pulse crop that provides an integral source of nutrition for human consumption, but production is hampered by Ascochyta blight. In lentil breeding programs, several Ascochyta blight resistance sources have been introduced into modern varieties, but their genetic basis and whether they are effective against the different pathotypes of Ascochyta blight are unclear. Here, we investigated the resistance in lentil line Indianhead by making a cross with the susceptible ILL6002. The offspring was inbred for six generations and subsequently evaluated with Pathotypes 1 and 2 in seedling assays and the field, and one major region in the lentil genome associated with Pathotype 1 resistance and an additional eight regions to either Pathotype 1 or 2 were identified. The markers flanking these regions in the genome will allow lentil breeding programs to trace the associated resistance in their breeding program pedigree, ensuring disease resistance is maintained in future varietal releases.

**Abbreviations**
AB: Ascochyta blight
HR: hypersensitive response
LOD: logarithm of odds
QTL: quantitative trait locus
PVE: percentage variance explained
RIL: recombinant inbred line

## 1 INTRODUCTION

Domestic lentil (*Lens culinaris* Medik) is a herbaceous and cool‐season annual pulse crop, part of the human diet for at least 58,000 years (Lev et al., 2005). High in dietary fiber and protein content, and well suited to crop rotation as they resist cereal diseases and fix soil nitrogen, lentils are grown and consumed throughout the modern world (Hall, 2018; Muehlbauer et al., 2002). Lentil cultivation worldwide is impacted by an array of biotic and abiotic stressors, of which disease caused by fungal pathogens is often the most impactful, both in terms of resulting damage and the cost of disease management.

Ascochyta blight (AB) is one such disease and is present in every major lentil‐growing region worldwide. AB of lentil results from infection by the hemibiotrophic fungal pathogen *Ascochyta lentis* Vassilievsky (teleomorph: *Didymella lentis*), which is host‐specific to members of genus *Lens* (syn. genus *Vicia* section *Lens*) (Omar et al., 2019; Schaefer et al., 2012). In Australia, AB is the most damaging and costly of lentil pathogens, and it is managed primarily using genetic resistance in tandem with cultural practices and fungicides (Murray & Brennan, 2012).

Recent studies conducted in Australia on domestic *A. lentis* isolates have identified two pathotypes with distinct patterns of infection on popular cultivars, each differentiated by which form of effector gene *AlAvr1* they carry (Henares et al., 2022; Henares et al., 2023). Isolates with *AlAvr1‐1* are “Pathotype 1” (“Nipper‐virulent”), and isolates with *AlAvr1‐2* are “Pathotype 2” (“Hurricane‐virulent”). In several popular Australian cultivars (PBA Bolt, PBA Hurricane XT, PBA Hallmark XT, PBA Herald XT), AlAvr1‐1 functions as an avirulence effector, triggering a hypersensitive response (HR) when recognized by the plant, which protects the host against infection and leads to Pathotype 1 AB resistance (Henares et al., 2022). Other Australian cultivars (PBA Ace and PBA Jumbo2) with stronger Pathotype 1 resistance are available and do not present with an HR when agroinfiltrated with either AlAvr1‐1 or AlAvr1‐2 (Grains Research & Development Corporation, 2024; Henares et al., 2022). Lentil cultivars Nipper, PBA Greenfield, and GIA Metro are more resistant to Pathotype 2, but the resistance and avirulence genes associated with this interaction are yet to be identified (Grains Research & Development Corporation, 2024; Henares et al., 2022; Thackwray et al., 2024).

In addition to managing the challenge of pathotype‐specific resistance, resistance genes may also occasionally become entirely non‐viable, such as previously “Moderately Resistant” cv. Laird, scored “susceptible” since the early 1990s, and Australian cultivars Northfield, Nugget, and PBA Flash, all downgraded around 2010 (Davidson et al., 2016; Kemal, 1996; Thackwray et al., 2024). Northfield served as a founding source of AB resistance to the Australian pedigree, alongside Canadian cultivar Indianhead. While Northfield resistance has been partially overcome, resistance from Indianhead has remained useful and has been used extensively in both Australian and Canadian breeding programs for this purpose.

Lentil is affected by over a dozen different fungal pathogens, with different species thriving, surviving, or completely absent from different regions worldwide. For example, powdery mildew of lentil (*Erysiphe* sp.) is typically a minor concern in most lentil‐growing regions, confined to recurrent infections in glasshouse environments where it may persist for years (Attanayake, 2008; Attanayake et al., 2009). It may, however, become highly damaging in instances of pathogen‐optimal environmental conditions. Areas reported as hotspots or sites of major damage by powdery mildew include Bihar, Madhya Pradesh, Rajasthan, and Uttar Pradesh in India, and Nahr an Nīl and Wilāyat al‐Kharṭūm in Sudan (Agrawal & Prasad, 1997; Food Legume Improvement Program, 1992; Padhy et al., 2023). In lentil, this disease results from infection by one of four biotrophic species from fungal genus *Erysiphe: E. diffusa* (Banniza et al., 2004)*, E. pisi* (Singh et al., 2012), *E. polygoni* (Akhtar et al., 2009), and *E. trifolii* (Attanayake et al., 2009), though lentil is not considered the primary host of any particular species.

Although less lethal to the host plant than many fungal diseases, powdery mildews are persistent ectopic parasites and may impact crop yield or leave the host susceptible to other pathogens. Early infection of lentil by *Erysiphe* sp. occurs in white spots or patches on the leaf surface, which may appear soft and powdery on closer inspection. The disease can be particularly damaging to seedlings or during flowering (Attanayake, 2008). Powdery mildew is not a target for major lentil breeding programs at present, and no quantitative trait locus (QTL) or specific resistance elements have been identified in response to mildew infection.

Following the degradation of previously deployed AB resistance‐associated genes in the Australian pedigree, developing a better understanding of already utilized and novel disease resistance genes is essential for maintaining effective resistance as the pathogen continues to evolve. To better understand the AB resistance present in cv. Indianhead, a hybrid population was developed from a cross with susceptible accession ILL6002. Two variants of ILL6002, one large seeded (AGG 74944) and one small seeded (AGG 74033), are cited in the literature. The small‐seeded stock of ILL6002 was crossed with Indianhead to develop a recombinant inbred line (RIL) population.

This population was evaluated for the response to Pathotypes 1 and 2 of *A. lentis* in both seedling assays and field experiments. In addition, the population was opportunistically assessed for the response to powdery mildew infection during a seed‐bulk event in the glasshouse. The QTLs identified herein, and markers associated with them, can be used as refined tools in lentil breeding programs to provide direction for more precise identification of associated resistance genes.

## 2 MATERIALS AND METHODS

### 2.1 Fungal isolates and plant material

*Ascochyta lentis* isolates P94‐24 and *Al*Kewell were received from the South Australian Research and Development Institute archive collection. P94‐24 was originally collected in Turretfield (South Australia) in 1996 and *Al*Kewell in Kewell (Victoria) in 2000. Field screen isolate WAE21002‐1 was originally collected in Esperance (Western Australia) in 2021, while WAC12393‐1 was received from the Western Australian Department of Primary Industries and Regional Development, originally collected in Merredin in 1998. Isolates P94‐24 and WAC12393‐1 are Pathotype 1, and isolates *Al*Kewell and WAE21002‐1 are Pathotype 2. Isolates P94‐24 and *Al*Kewell were nominated for controlled environment screens as they are the facility reference standards for Pathotypes 1 and 2, and were substituted for isolates WAC12393‐1 and WAE21002‐1 under field use as Western Australian collections minimize the risk of interstate genetic spread and the introduction of potentially novel *A. lentis* isolates to the immediate area.

**Core Ideas**

- Generated a lentil recombinant inbred line population derived from cultivar Indianhead and accession ILL6002 to evaluate for Ascochyta blight resistance.
- Eight distinct loci effective against Pathotype 1 and Pathotype 2 were identified from seedling and field assays for Ascochyta blight resistance.
- A natural powdery mildew infection was scored, and quantitative trait locus (QTL) analyses identified a major QTL on chromosome 3.

Parental lentil seed was similarly used from stocks of bulked seed stored at the CCDM, originally obtained from the Australian Grains Genebank. Control accessions Cumra, ILL7537, Nipper, PBA Bolt, PBA Greenfield, and PBA Hurricane XT were received from the national lentil breeding program at Agriculture Victoria (AgVic).

### 2.2 Population development

An F_1_ hybrid of resistant cv. Indianhead and the susceptible accession ILL6002 was generated with manual crossing procedures and progressed by single seed decent to generate an F_6_ RIL population. Indianhead was selected as the pollen donor, and ILL6002 was selected as the seed parent. True hybrids were identified by seed coat color of the F_2_ seeds produced by the F_1_ hybrid (Figure S1). F_1_ individuals were grown under winter glasshouse conditions with no supplementary lighting to produce approximately 300 F_2_ seeds. F_2_ individuals were grown in a field with deep sandy soil under natural winter conditions, supplemented with additional irrigation in the spring. Individuals from the F_3_ and F_4_ generations were grown in a controlled environment room under a 16/8 h photoperiod with artificial lighting, with temperatures ranging between 18°C and 23°C. Seeds were sown at a depth of 1 cm in 125 mm 1‐L plastic pots containing UWA multi‐purpose potting mix (Richgro). Plants were watered as required and fertilized intermittently with Nitrophoska Perfect NPK fertilizer.

Multiple seeds of F_5_ individuals were started under winter glasshouse conditions and sown in 200 mm 4‐L plastic pots. Following extensive spread of powdery mildew under glasshouse conditions, plants were scored for visible infection (detailed below) and moved to open‐air conditions under natural winter weather and treated with AMISTAR 250 SC fungicide to resolve the powdery mildew infection. Plants were fertilized intermittently with Nitrophoska Perfect and watered as required. Seed from 186 F_6_ RIL accessions was obtained. Accessions lost between F_2_ and F_6_ were due to repeated failure to germinate or reoccurring premature death. A simplified illustration of the environments for each generation is provided in Figure S2.

### 2.3 Genotyping of the RIL population

Leaf material (80–110 mg) from 186 F_5_ individuals was sampled into 1.2 mL collection microtubes and subsequently freeze‐dried over a period of 48 h. The prepared samples were then sealed, labeled as appropriate, and sent to GrainDataGen (Agriculture Victoria) for genotyping using the multispecies pulse 30K single‐nucleotide polymorphism (SNP) chip, containing 10,528 lentil‐specific SNPs (Sudheesh et al., 2023). In addition to the 186 RIL individuals, material from both parents was included in duplicate as both a reference, and an internal control. F_6_ seed suitable for phenotypic disease screening was obtained for 168 of these 186 individuals.

Genomic DNA extraction was performed using MagMAX Plant DNA Kit (Thermo Fisher Scientific Inc.), and the concentration and quality of the DNA were confirmed by a NanoDrop 8000 spectrophotometer (Thermo Fisher Scientific Inc.). For each sample, 200 ng gDNA was used for the genotyping on the Illumina multispecies pulse 30K SNP chip. Data analyses were performed using GenomeStudio 2.0 Polyploid software (Illumina) using the lentil SNP manifest file. Theta and normalized *R* values were exported and used to call SNPs as described by Gebremedhin et al. (2024).

### 2.4 Powdery mildew phenotyping

Phenotyping for powdery mildew infection was performed opportunistically following inadvertent natural infection of the complete F_5_ population (*n* = 186) under glasshouse conditions. Plant age averaged 50 days post‐germination at time of scoring, with all individuals in either inductive or post‐inductive vegetative growth states. Scoring was limited to the adaxial leaf surface, as mildew infection occurs more frequently adaxially, and as lentil adaxial trichomes tend to be both longer and less dense than on the abaxial, limiting any risk of confusion with early mildew infection (Patel et al., 2021). All adaxial leaf surfaces of each plant were examined with the naked eye for evidence of powdery mildew and scored for presence‐absence with values as “Present” (= 1) where observed and “Absent” (= 0) where not observed. Two plants for each accession were examined. Binary scoring was nominated for powdery mildew evaluation, as mildew resistance and susceptibility are often qualitative traits in other systems and similarly scored as such (Holdsworth et al., 2016; Poulter et al., 2003; Stack et al., 2024).

### 2.5 AB seedling assay

Phenotyping for AB was conducted using adjustments on established protocols described in Henares et al. (2022). Three repeats of three seedlings per punnet for each RIL accession were sown in a randomized complete block design, with wheat used to border each punnet to encourage plant stability and consistent humidity. A total of 168 F_6_ RIL accessions were used for seedling assays. Included as controls were both parents and four additional lentil lines with different levels of AB resistance and pathotype response. ILL7537 is highly resistant regardless of pathotype and served as a resistant control. PBA Hurricane XT is highly resistant to Pathotype 1 and susceptible to Pathotype 2. Conversely, Nipper and PBA Greenfield are both resistant to Pathotype 2 and susceptible to Pathotype 1.

The RILs, parents, and differential lentil lines were screened with both pathotypes, with isolates P94‐24 and *Al*Kewell used to represent Pathotypes 1 and 2, respectively. *Ascochyta lentis* spores from frozen glycerol stocks were grown at room temperature (20.0°C ± 2.0°C) under long‐wave UV light with a 12/12 photoperiod on plates of ½ PDA (Potato Dextrose Agar) with 0.1% streptomycin. *Ascochyta lentis* culture plates were prepared 5, 7, and 9 days post‐sowing to ensure availability of mature conidia on the day of infection. Plates were flooded with autoclaved water to harvest spores from mature pycnidia at about 13 days post‐sowing and filtered through sterile cotton wool. Once the majority of seedlings had grown to the sixth node, or four fully open leaves (13 ± 1 days after planting), seedlings were spray‐inoculated with suspensions of *A. lentis* spores at a concentration of 1 ± 0.2 × 10^6^ spores/mL, with 0.1% Tween 20, in autoclaved water.

Inoculated plants were grown under a 12/12 photoperiod, with temperatures maintained between 18°C and 23°C. Plants were misted for 5 s every 4 h for the first 2 days following infection, decreased to 5 s every 6 h for 8 days, and finally, every 12 h for 4 days. Misting was turned off, wheat was cut back, and lentil was allowed to dry overnight prior to scoring at 14 days post‐infection. Plants were scored for infection using percentage leaf area damage (LAD), with scores at increments of 5% (Henares et al., 2019).

### 2.6 AB field phenotyping

To assess resistance under field conditions, field plots were planted at Northam (Western Australia, 31°39′2″ S, 116°41′52″ E) in the 2024 winter growing season. Local substrate is red, loamy soil, and rhizobium inoculant (group E; *Rhizobium leguminosarum* bv. *viciae* strain WSM4643 [Alosca Technologie]) was provided. RIL population plants at the F_6_ (*n* = 104) were grown under natural weather conditions, supplemented with irrigation as required. Duplicate experiments were planted to accommodate separate screening with each *A. lentis* pathotype. Within experiments, accessions were planted in triplicate, with a minimum of five seeds to each plot in a random block design. Rows were sown 22 cm apart, and plots were planted 20 cm apart within each row. Plots were interspersed with susceptible cv. Cumra to encourage further AB infection and spread once pathogen inoculation had occurred. Due to seed availability, only 104 of the 186 RIL accessions were used for these screening experiments. Included as controls for field screening were parents ILL6002 and Indianhead, ILL7537, Pathotype 2‐resistant Nipper, and Pathotype 1‐resistant PBA Bolt, which resembles PBA Hurricane XT very closely in pathotype response but is better suited to the local soil, owing to improved salinity and boron tolerances.

Isolates WAC12393‐1 and WAE21002‐1 were selected to represent Pathotype 1 and Pathotype 2, respectively. Plants were initially infected 61 days post‐sowing, prior to flowering but following canopy establishment. Initial inoculation utilized liquid spore suspension and was applied to experiments for both pathotypes. Liquid spore suspension was prepared as described previously, but to a concentration of 0.5 × 10^6^ spores/mL.

Seventeen days after the spore suspension inoculation, the field plots were inoculated again using wheat (*Triticum aestivum* L.) grain inoculum of isolates. Wheat grain inoculum was prepared using 1‐ and 2‐L bags of wheat seed, sterilized and inoculated with similarly sterile spore suspensions of *A. lentis* isolates WAE21002‐1 and WAC12393‐1. *Ascochyta lentis* was allowed to grow until sufficient mycelial growth was established (approximately 1–2 weeks), then dried at room temperature and coarsely ground before use. Ground wheat inoculum was applied to plots at 20 g/m^2^.

The WAE21002‐1 (Pathotype 2) infected plants were scored for disease 48 days after first inoculation, and the WAC12393‐1 (Pathotype 1) infected plants were scored 59 days after first inoculation. Field scoring utilized a disease incidence rating, scaled between 0 and 5 on visible LAD, where 0  =  no disease infection; 1  =  1%–20% LAD; 2  =  21%–40% LAD; 3  =  41%–60% LAD; 4  =  61%–80% LAD; and 5 = 81%–100% LAD.

### 2.7 Statistical analysis

Mean replicate AB disease scores for each RIL accession per AB experiment were generated and standardized to receive *z*‐scores. *z*‐Scores were generated by experiment and were chosen over other transformation methods as they allow for the accurate and appropriate collation of data between experimental replicates. An individual *z*‐score (𝑧) was generated with $z=\frac{x-\mu }{\sigma }\;$, where 𝑥 = raw score, *μ* = population mean, and *σ* = population standard deviation, with calculations for this data performed in Microsoft Excel. Scores for powdery mildew were not transformed.

To investigate potential correlation between the phenotypic results of each experiment, Spearman's rank correlation was employed, using the cor.test function available with R package R/stats (R Core Team, 2018).

### 2.8 Linkage map construction

Genetic linkage maps were constructed with R‐based package R/ASMap (Taylor & Butler, 2017). Markers received from genotyping of the F_5_ population were filtered to remove monomorphic instances, markers missing ≥15% of values, and those with genotype frequency ≥85% to account for the effects of segregation distortion. The filtered markers were then clustered and ordered within linkage groups (LGs), using the ASMap function MSTmap. Parameters used were distance function (dist.fun) “Kosambi”, population type (pop.type) RIL5, and *p*‐value (*p*.value) of 0.9 × 10e^−9^. Unlinked markers and minor LGs (<10) were then discarded, and the resulting map was examined further with ASMap functions “profileGen” and “profileMark” to remove markers showing double crossovers, missing data, and remaining segregation distortion caused by high heterozygosity. After filtering, the map was reconstructed and evaluated visually using an ASMap‐produced heatmap, which gives estimates of logarithm of odds (LOD) scores between marker pairs and pairwise recombination.

### 2.9 QTL analysis

The analyzed population consisted of all 186 genotyped F_5_ RIL lines for powdery mildew, the 168 F_6_ RIL lines phenotyped for AB seedling assay, and 104 F_6_ RIL individuals for AB field screening. QTL analysis was conducted with R‐based package R/qtl2, using Haley–Knott regression for AB resistance and logistic regression for the binary presence‐absence scores taken for powdery mildew infection (Broman et al., 2019). The permutation test in R/qtl2 with 1000 iterations was used to determine LOD significance threshold (*p *< 0.05), with statistical boundaries determined with a 1.5 support interval. To confirm the significance of markers linked to the identified QTL, Kruskal–Wallis rank sum testing was employed, using the kruskal.test function available in R/stats (R Core Team, 2018). Kruskal–Wallis rank sum is considered the appropriate non‐parametric alternative to one‐way ANOVA.

QTL nomenclature was devised based on previous studies (McCouch & THE Committee on Gene Symbolization‚ Nomenclature and Linkage, 2008; Boden et al., 2023). Name construction was created in sequence, as follows: (1) the letter “q”; (2) “AB” or “PM,” to identify AB and powdery mildew, respectively; (3) an underscore; (4) the numerical chromosome or LG the QTL was identified on; (5) a period; (6) a second number, assigned in sequence to distinguish multiple QTL identified on the same chromosome or LG. Once the boundaries of the QTL regions were identified on respective LGs, the analogous regions were then identified on the CDC Redberry (v2.0) genome, and annotations for genes within each region were catalogued. Particular note was given to the catalogued plant defense‐associated genes, which included both conventional resistance gene analogues (RGAs) and other genes commonly involved with resistance machinery, such as calcium signaling components, ethylene response factors (ERF), glutamate receptors, jasmonate (JA) ZIM domain proteins, NBS‐LRR genes, pentatricopeptide repeat‐containing (PPR) genes, protein kinases, transmembrane proteins, and zinc‐finger‐containing genes. Additionally, markers from previous studies identifying AB resistance QTL in Indianhead and progeny with resistance attributed to Indianhead were remapped to the CDC Redberry (v2.0) genome assembly to identify where any resistance genes may be associated with those previously observed.

## 3 RESULTS

### 3.1 Development of an ILL6002 × Indianhead RIL population and associated genetic map

Cultivar Indianhead has been a popular parent in both Australian and Canadian lentil breeding programs as it harbors a useful source of AB resistance. To dissect the resistance and determine respective effectiveness against Pathotype 1 and 2 isolates, an RIL population (*n* = 186) was generated from lentil lines Indianhead and ILL6002.

The 186 RILs were genotyped using the multispecies pulse 30K SNP chip, containing 10,528 lentil‐specific SNP markers (Sudheesh et al., 2023). A total of 10,244 SNPs were obtained, with 3193 polymorphic SNPs between parents Indianhead and ILL6002 (30.33% of markers on the SNP chip). The highest value for missing SNP marker data for the RIL accessions was 6.75%, so no accessions were excluded on the basis of missing data. Markers with >15% missing data and markers that could result in segregation distortion due to high heterozygosity or genotype frequency were omitted, and the 2662 remaining markers (Table S1) were used to construct a genetic map, which spanned a total of 2117 cM, with an average distance between markers of 0.80 cM (Figure S3; Table 1). The exact number of markers removed at each point in the quality control process is available in Table S2.

**TABLE 1:** Marker count, length, and mean distance between markers for constructed ILL6002 × Indianhead linkage groups.

Linkage group	Marker count	Length (cM)	Mean distance between markers (cM)
**LG1**	306	224.3	0.74
**LG2**	516	461.9	0.90
**LG3**	443	320.9	0.73
**LG4**	378	341.6	0.91
**LG5**	418	199.7	0.48
**LG6**	230	394.9	1.70
**LG7**	371	173.3	0.47
**All**	2662	2117	0.80

Figure S4 shows a heatmap with the recombination rates between each marker on the seven LGs. The shortest LG was LG7, at 173.3 cM, and the longest was LG2, at 461.9 cM. LG6 had the fewest markers (*n* = 230), and LG2 had the most (*n* = 516). A comparison between the constructed linkage map and provided locations for each marker on the CDC Redberry (v2.0) genome assembly indicated marker order was largely synonymous between the two. The distribution and comparison of location of SNPs across the seven LGs and position on respective chromosomes are presented in Figure S5. In the comparison of LGs with the physical map of the CDC Redberry (v2.0) reference genome, no translocations were observed, but large inversions were observed centrally on chromosomes 2 and 6, and a small inversion central to chromosome 7, as well as minor marker relocations on chromosomes 3, 4, and 5 (Figure S5).

### 3.2 Evaluation of the RIL population for resistance to AB

The RIL population was evaluated for their response to AB infection in seedling assays and in field conditions. For seedling assays in controlled environment conditions, P94‐24 (Pathotype 1) and *Al*Kewell (Pathotype 2) were used. For AB field screens, Western Australian isolates WAC12393‐1 (Pathotype 1) and WAE21002‐1 (Pathotype 2) were used, deemed more appropriate than interstate collections due to interstate quarantine precautions.

**FIGURE 1:**
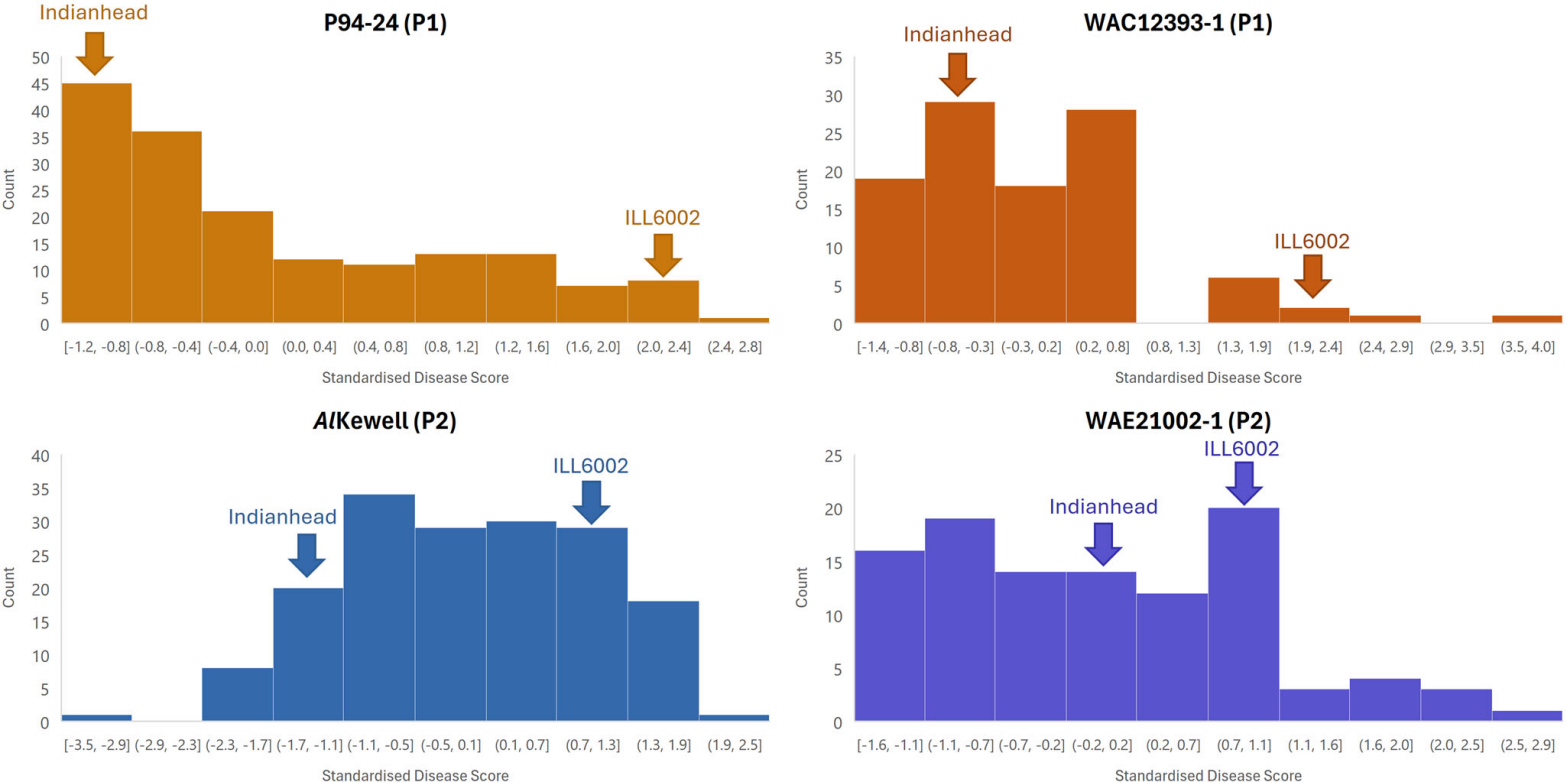
Frequency distribution of the ILL6002 × Indianhead recombinant inbred line (RIL) population standardized means of disease screens for each individual, recorded for each isolate. Larger *z*‐scores indicate higher recorded disease symptoms within the population. The mean value recorded for either population parent is indicated with a labeled arrow. Isolates marked P1 are Pathotype 1, and isolates marked P2 are Pathotype 2. Seedling assays are presented on the left, and field assays are presented on the right.

Scores underwent *Z*‐standardization prior to examination to allow for accurate collation between replicates and to greater enhance the ease of comparison between separate experiments. Parent Indianhead was found to have greater resistance than parent ILL6002 in all experiments, and to a greater degree under infection by both of the Pathotype 1 isolates compared to the Pathotype 2 isolates (Figure 1). Distribution of standardized phenotypic data demonstrated differing patterns of resistance within the population for each isolate. A Shapiro–Wilk test confirmed non‐normal distribution of means for all isolates in the respective experiments (*p*‐values: P94‐24 = 7.2 × 10^−10^, *Al*Kewell = 0.036, WAC12393‐1 = 1.27 × 10^−5^, WAE21002‐1 = 0.0022). The frequency distribution of disease scores ranged from unimodal skewed strongly toward lower disease scores for P94‐24 to unimodal with a broadly centralized plateau for *Al*Kewell (Figure 1). Accessions more resistant and more susceptible than either parent were noticeably present for Pathotype 2 isolates *Al*Kewell and WAE21002‐1, suggesting transgressive segregation. A Dunnett's test conducted between standardized parental means and those of individual RIL lines was used to further investigate the distribution of disease response phenotypes but could not confirm any significant differences from the nearest parent in any measured individuals. Greater disease severity was observed in the population with infection by Pathotype 2 isolates *Al*Kewell and WAE21002‐1 than their respective Pathotype 1 counterparts, P94‐24 and WAC12393‐1. Spearman's rank correlation testing between phenotypic scores for each experiment found weak but statistically significant positive correlation between Pathotype 1 isolates P94‐24 and WAC1293‐1 (Rho = 0.265, *p*‐value = 0.006), and between Pathotype 2 isolates *Al*Kewell and WAE21002‐1 (Rho = 0.237, *p*‐value = 0.014) (Table S3). Interestingly, a weak but similarly significant negative correlation (Rho = ‐0.213, *p*‐value = 0.029) was observed between isolate WAE21002‐1 and powdery mildew infection phenotyping.

Further transformation of the Z‐standardized data in the pursuit of normality was examined, but transformations either did not improve normality or did not provide any notable differences in the resulting analysis from the already‐standardized data, so they were not used. To support the results, QTLs were verified using Kruskal–Wallis rank‐sum testing.

### 3.3 QTL analysis for AB resistance identifies eight QTL

**FIGURE 2:**
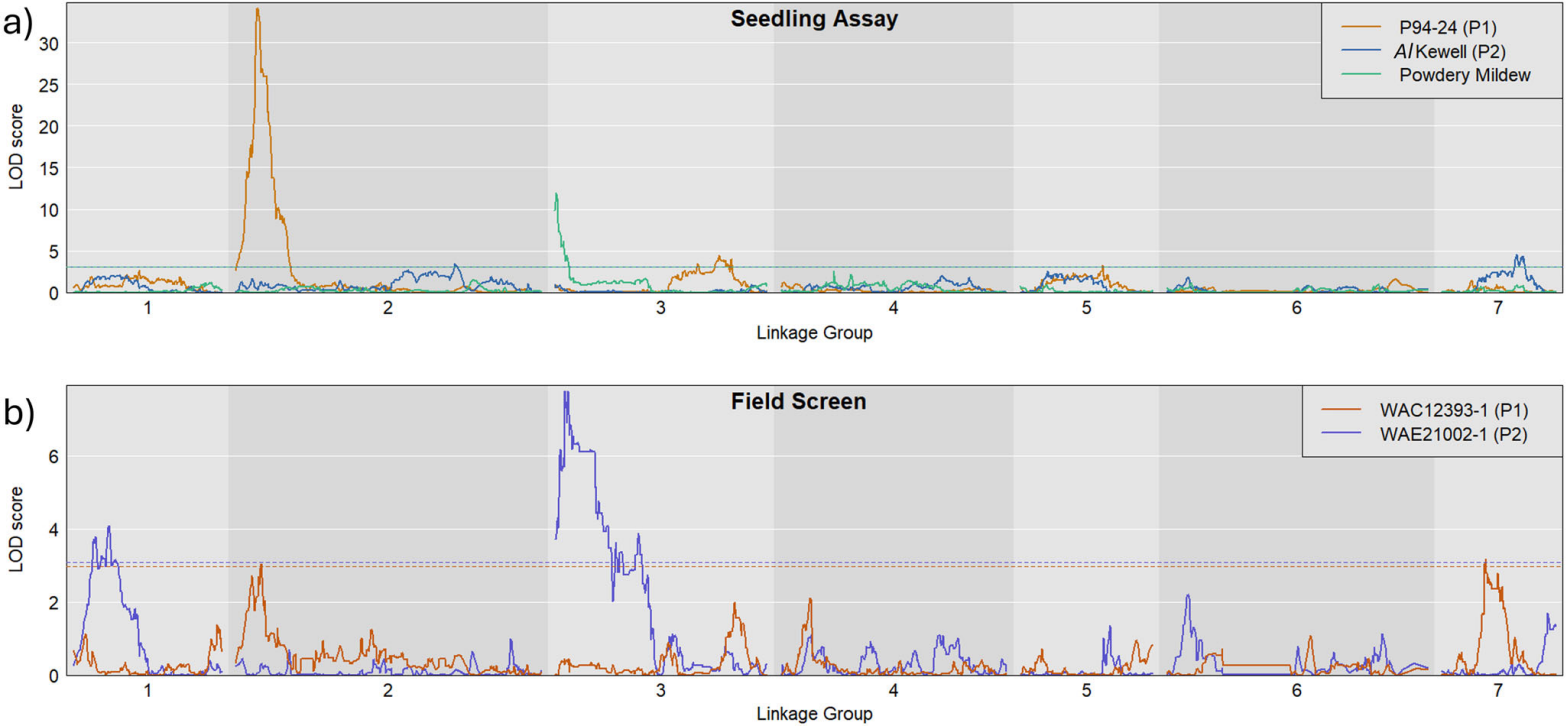
Quantitative trait locus (QTL) analysis of (a) Ascochyta blight seedling disease assays (*n* = 168) and (b) field screens (*n* = 104) conducted using the ILL6002 × Indianhead recombinant inbred line (RIL) population and constructed linkage maps. Isolates noted P1 are Pathotype 1, and isolates noted P2 are Pathotype 2. Horizontal lines indicate the logarithm of odds (LOD) score significance (*p*‐value < 0.05), determined using a 1000‐iteration permutation test.

QTL analyses of field and seedling phenotypic screens (Table 2) identified a total of eight unique QTL, with the boundaries, and therefore the size of each, as determined by the analysis itself (Table 2; Figure 2). All QTLs were confirmed significant using Kruskal–Wallis rank sum testing. One QTL present at the same locus in multiple screens was observed, designated qAB_2.1. The QTL qAB_2.1 was observed in response to Pathotype 1 isolates P94‐24 (LOD = 34.1) and WAC12393‐1 (LOD = 3.0), explaining 60.5% and 12.6% of phenotypic variance, respectively, where the resistance is inherited from the Indianhead parent. The corresponding region in the CDC Redberry (v2.0) genome assembly associated with qAB_2.1 is 2.6 Mbp in width and contains 99 annotated genes, 21 of which are annotated as plant defense‐associated genes and three as resistance gene analogues (Table 2).

**TABLE 2:** Significant quantitative trait locus (QTL) identified on constructed linkage groups of the ILL6002 × Indianhead recombinant inbred line (RIL) population, and their associated region on the CDC Redberry (v2.0) genome.

					Linkage group				CDC Redberry (v2.0) genome									
QTL	Disease	Isolate	Pathotype	Chr/LG	Lod score	Lower boundary	Peak (cM)	Upper boundary	Lower boundary	Peak (Mbp)	Upper boundary	ROI size (Mbp)	Genes	PDAG	RGAs	KW test (*P*)	Effect size	% Explained
**qAB_2.1**	AB	P94‐24	1	2	34.1	29.1	32.0	36.1	6.8	7.8	9.4	2.6	99	21	3	<2.2×10^−16^	0.80	60.5%
**qAB_3.1**	AB	P94‐24	1	3	4.5	213.5	249.0	268.7	349.3	376.1	405.1	55.8	1119	129	15	3.71×10^−5^	0.37	11.5%
**qAB_5.1**	AB	P94‐24	1	5	3.3	48.8	124.6	133.5	26.4	443.9	457.4	431.0	5666	656	104	1.52×10^−3^	0.29	8.5%
**qAB_2.2**	AB	*Al*Kewell	2	2	3.4	251.1	331.9	348.7	450.3	459.6	465.8	15.4	204	21	1	4.35×10^−5^	−0.34	8.8%
**qAB_7.1**	AB	*Al*Kewell	2	7	4.6	110.8	113.9	128.7	501.6	506.8	514.5	12.9	455	51	2	6.39×10^−3^	−0.35	11.5%
**qAB_2.1**	AB	WAC12393‐1	1	2	3.0	11.5	38.8	45.8	1.6	11.2	12.3	10.7	487	96	24	3.52×10^−3^	0.17	12.6%
**qAB_7.2**	AB	WAC12393‐1	1	7	3.2	61.6	67.1	99.9	28.3	55.5	486.8	458.5	5394	566	67	5.48×10^−3^	0.18	13.1%
**qAB_1.1**	AB	WAE21002‐1	2	1	4.1	22.4	53.0	71.1	9.5	27.1	40.2	30.7	493	58	4	1.63×10^−5^	−0.36	16.8%
**qAB_3.2**	AB	WAE21002‐1	2	3	7.8	14.9	15.1	37.2	19.0	21.4	81.7	62.7	985	166	87	8.74×10^−5^	0.48	29.6%
**qPM_3.1**	PM	–	–	3	11.8	0.0	1.7	5.2	2.3	4.8	6.9	4.6	92	10	5	5.75×10^−10^	−1.72	24.9%

*Note*: QTL boundary locations determined using a 1.5‐LOD support interval, logarithm of odds (LOD) score significance determined using a 1000‐iteration permutation test. Genes and resistance gene analogues (RGAs) as annotated on the CDC Redberry (v2.0) genome assembly (Ramsay et al., 2021). A detailed list of genes within each region, including plant defense‐associated genes (PDAG) and RGAs, can be accessed in file Supplementary_Data_1.xlsx. Positive effect size indicated resistance was associated with cv. Indianhead, and negative effect size indicated resistance was associated with ILL6002.

Abbreviations: AB, Ascochyta blight; Chr, chromosome; KW test (*p*), Kruskal–Wallis rank sum test *p*‐value; LG, linkage group; PM, powdery mildew; ROI, region of interest.

The two additional QTLs, designated as qAB_3.1 and qAB_5.1, observed in response to the inoculation of seedlings with Pathotype 1 isolate P94‐24 were associated with parent Indianhead. QTL qAB_3.1 is located on LG3 with a LOD score of 4.5, whereas qAB_5.1 is located on LG5 with a LOD score of 3.3. The three combined QTL effective against Pathotype 1 isolate P94‐24 explain 80.5% of the phenotypic variance for AB resistance, with qAB_3.1 and qAB_5.1 explaining 11.5% and 8.5%, respectively. The qAB_3.1‐corresponding region of interest on CDC Redberry chromosome 3 contains 1119 annotated genes, 134 of which are plant defense associated, including 15 RGAs. The qAB_5.1 corresponding region on chromosome 5 of the CDC Redberry genome is 431.0 Mbp in width, and contains 5666 genes, including 104 RGAs.

AB disease assays of lentil seedlings with Pathotype 2 isolate *Al*Kewell identified two QTL. Designated qAB_2.2 and qAB_7.1, resistance at both of these loci is associated with parent ILL6002. QTL qAB_2.2 is distal from major locus qAB_2.1 on LG2 with a LOD score of 3.4, explaining 8.8% of the variance, whereas qAB_7.1 is located on LG7 with a LOD score of 4.6, explaining 11.5% of the AB phenotypic variance. The corresponding regions on chromosomes 2 and 7 contain 204 and 455 annotated genes, respectively.

QTL analysis using the field phenotype data for Pathotype 1 isolate WAC12393‐1 resulted in qAB_2.1, also identified from the seedling assays discussed above, and qAB_7.2, observed on LG7 with an LOD score of 3.2 and PVE (percentage variance explained) of 13.2%. Resistance at both loci was associated with parent Indianhead, and combined explained 25.7% of the variance.

Lastly, QTL analysis using the field evaluation data with Pathotype 2 isolate WAC21002‐1 identified qAB_1.1 and qAB_3.2. QTL qAB_1.1 is on LG1, with a LOD score of 4.1 and a PVE of 16.8%, and is associated with ILL6002 with an effect size of −0.36. QTL qAB_3.2 was identified on LG3 with a LOD score of 7.8, explaining 29.6% of the AB resistance phenotype, which is associated with Indianhead. In summary, eight unique QTLs were identified. Resistance to Pathotype 1 isolates was inherited exclusively from the Indianhead parent, whereas Pathotype 2 resistance was associated with loci from both parents (Table 2). Boxplots presenting disease resistance scores within the RIL population as they relate to the parental alleles at the most favorable markers for each QTL are presented in Figure S6.

### 3.4 Opportunistic screening of a powdery mildew outbreak results in one major QTL

An outbreak of powdery mildew occurred in the glasshouse during the F_5_ seed bulk and was opportunistically scored (Table S4). Each individual in the RIL population was assessed and scored visually for presence or absence of disease symptoms. Of the 186 individuals, 39 showed clear symptoms, 145 did not show visible symptoms, and two were uncertain. Chi‐square testing found this ratio differed significantly from the 1:1 ratio that would be expected of a single major gene at homozygosity (χ^2^ = 61.065, *p*‐value = 5.521 × 10^−15^), but did not differ significantly from the 1:3 ratio expected of two major genes (χ^2^ = 1.420, *p*‐value = 0.233). However, as the scoring was conducted opportunistically and under greenhouse conditions, the numbers may not accurately reflect the total number of susceptible individuals. Subsequent QTL analysis for the presence of powdery mildew infection identified a single major QTL at the start of LG3, confirmed with Kruskal–Wallis rank‐sum. The QTL, designated qPM_3.1, had an LOD score of 11.8 with an effect size of −1.72, thus associated with resistance in parent ILL6002, and explained 24.9% of the variance observed. The corresponding qPM_3.1 region of interest in the CDC Redberry genome contains 92 annotated genes, 10 of which were plant defense‐associated genes, including five RGAs, but no MLO homologues (Figure S7).

## 4 DISCUSSION

A RIL population was developed from a cross of AB‐resistant lentil cultivar Indianhead and the susceptible accession ILL6002 to identify the key loci involved in the AB resistance response. Using the multispecies SNP chip (Sudheesh et al., 2023), a high‐quality genetic map comprising 2662 markers was generated (Table 1), and comparison to the reference lentil genome identified inversions on chromosomes 2, 6, and 7, but no major genomic rearrangements in either parent.

Although early lentil linkage maps were occasionally used in tandem with Indianhead (Andrahennadi, 1994; Chowdhury et al., 2001), previous linkage maps created using the cultivar have been published in just one study (Sudheesh et al., 2016). Two maps, based on hybrids with Australian cultivars Northfield (ILL5588) and Digger (ILL5722), were constructed for the purpose of mapping AB resistance in each of the three cultivars used, using 460 and 329 markers, respectively, across seven LGs corresponding to the initial CDC Redberry genome assembly (Sharpe et al., 2013; Sudheesh et al., 2016). The length of these LGs was estimated at 1461.6 cM for Indianhead × Northfield and at 1302.5 cM for Indianhead × Digger, an average of 3.18 and 3.96 cM between markers on either map, respectively. The map presented here improves on this in marker density, with a 0.8 cM average. While modern technologies allow for the easy development of superior linkage maps with as little as 0.1 cM on average between markers, the 30K SNP chip for pulses was nominated for use in this study as it is cost‐effective, ensures reliability for the repetitive lentil genome, allows for easy comparison with the current most comprehensive genome assembly, CDC Redberry (v2.0), and other studies utilizing the same resource. Perhaps most notably, the 30K SNP chip is also in use by the Australian lentil breeding program, providing the benefit of immediate marker compatibility with existing industry resources.

**FIGURE 3:**
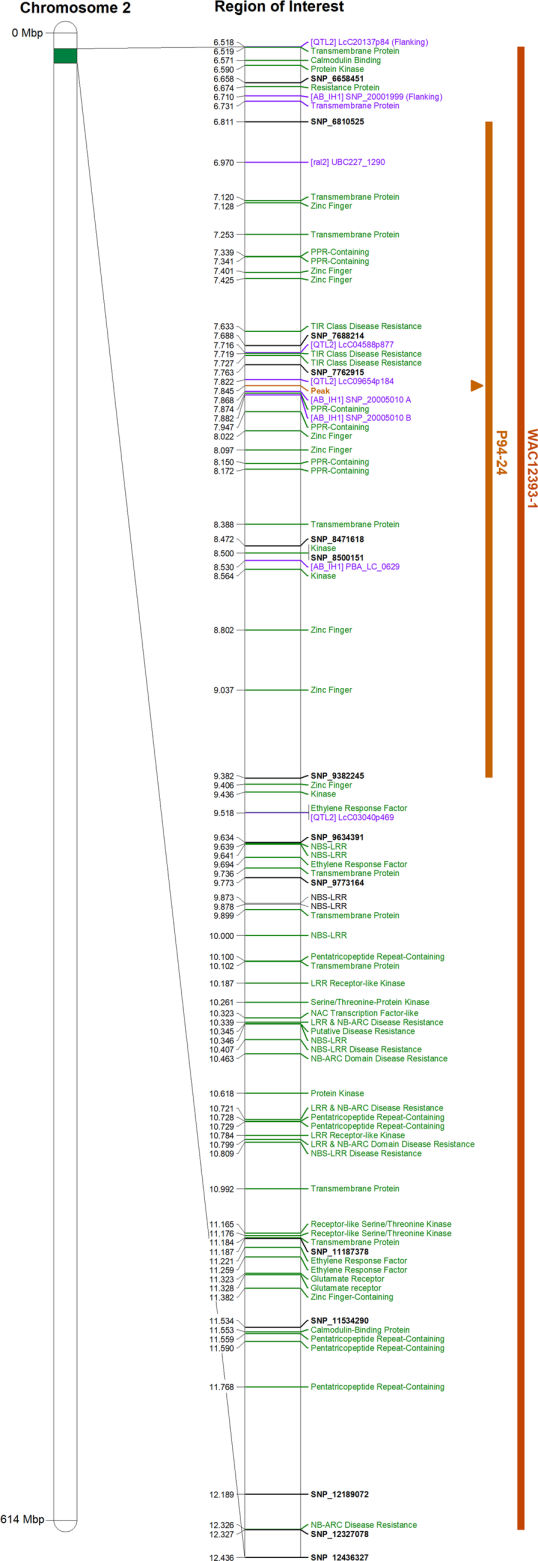
The region under quantitative trait locus (QTL) qAB_2.1 as seen in response to isolates P94‐24 and WAC12393‐1. The red and orange bars indicate the spans of the QTL, with the peak marked on the bar. Single‐nucleotide polymorphisms (SNPs) originate from the multispecies pulse SNP chip (Sudheesh et al., 2023) and are noted in black. Resistance gene analogues are as annotated on the CDC Redberry (v2.0) genome assembly and noted in green. Markers associated with resistance QTL from previous studies are noted in purple (Sari, 2014; Sudheesh et al., 2016; Tar'an et al., 2003).

Examination of the putative resistance loci present in the ILL6002 × Indianhead RIL population identified multiple QTL associated with resistance to AB, but just one reoccurring QTL. QTL qAB_2.1, located at the start of LG2, and a corresponding position on chromosome 2, occupied a narrow region of 3.2 Mbp with a LOD of 34.1 when screened with P94‐24 (Pathotype 1) under controlled conditions and a less dramatic but still noteworthy equivalent with a LOD of 3.0 when screened with WAC12393‐1 (Pathotype 1) under field conditions. Spearman's rank correlation testing found a weak but statistically significant correlation between the phenotypic scores of both Pathotype 1 isolates, possibly reflecting the presence of this QTL in both isolates. This QTL is of note for several reasons. The first is that both isolates carry the avirulence gene *AlAvr1‐1*, thus continuing an observed trend of resistance in Indianhead and descendants to resist Pathotype 1 isolates more strongly than Pathotype 2 (Henares et al., 2022; Sudheesh et al., 2016). Second, qAB_2.1 sits in a genomic region rich in plant defense‐related genes, 11 times higher than the predicted genome‐wide average density of such genes (Guerra‐Garcia et al., 2022). Under field conditions, the statistical boundaries of qAB_2.1 on the CDC Redberry (v2.0) reference assembly contain 24 RGAs, which decreased to just three when seedlings were screened in a controlled environment. Lastly, the remapping of markers associated with three previous studies on Indianhead and CDC Robin were found to closely flank the region, suggesting qAB_2.1 may contain the gene, or genes, previously identified with AB_IH1 (Sudheesh et al., 2016), QTL2 (Sari, 2014), and ral2 (Tar'an et al., 2003) (Figure 3; Table 3). CDC Robin is the genetic grandchild of Indianhead by way of CDC Matador, and resistance in CDC Robin is frequently attributed to Indianhead (Sari et al., 2017; Tar'an et al., 2003; Vandenberg et al., 2002).

**TABLE 3:** Previously documented resistance as identified in cv. Indianhead. Also included is CDC Robin, as Indianhead is considered to be the source of Ascochyta blight (AB) resistance in CDC Robin.

Study	ID	Mapping population	*Ascochyta lentis* Isolate	Plant age at initial infection	Assessed	Mode of inheritance
Sudheesh et al. (2016)	AB_IH1	Indianhead/Digger Indianhead/Northfield	FT12013 FT13038	14 Days	14 DPI	Recessive
Sudheesh et al. (2016)	AB_IH2.1	Indianhead/Northfield	FT13038	14 Days	14 DPI	Recessive
Sudheesh et al. (2016)	AB_IH2.2	Indianhead/Digger	FT12013	14 Days	14 DPI	Unclear
Sari (2014)*	QTL2	CDC Robin*/964a‐46	AL56	21 Days	14–21 DPI	Recessive
Tar'an et al. (2003)*	ral2	CDC Robin*/964a‐46	A1 3D2	28 Days	14 Days	Recessive
Ye et al. (2003)	Abr1	Indianhead/Laird Indianhead/Titore	Rakaia	7th Node	7–21 DPI	Recessive with additive effects
Ye et al. (2003)	Abr2	Indianhead/Laird Indianhead/Titore	Rakaia	7th Node	7–21 DPI	Recessive with additive effects
Andrahennadi et al. (1994), Chowdhury et al. (2001)	ral2	Indianhead/Northfield Indianhead/Eston	Unknown	40 Days	15 DPI	Recessive

Abbreviation: DPI, days post inoculation.

Markers for QTL AB_IH1 recorded by Sudheesh et al. (2016) can be traced through the pedigree of Australian cultivars from Indianhead to more recent descendants such as PBA Hurricane XT and PBA Bolt, correlating closely with the presence of improved Pathotype 1 resistance (Henares et al., 2022; Sudheesh et al., 2016; Thackwray et al., 2024). All cultivars in the Australian pedigree demonstrating greater resistance to Pathotype 1 are descendants of Indianhead, although as Indianhead does not demonstrate the same HR to AlAvr1‐1 as is seen in PBA Hurricane XT, PBA Bolt, and similar cultivars when subjected to agroinfiltration, the association remains in question (Henares et al., 2022). Whether any of the QTL identified herein are interacting with AlAvr1‐1 remains unclear, and further genetic studies are warranted.

While markers for qAB_2.1 correlate closely with previous studies, the region identified here is narrower than previous investigations, which should allow for greater accuracy of selection in future breeding efforts and confirm that this locus is only effective against Pathotype 1 isolates. Fine mapping and Nanopore adaptive resequencing would be useful for further investigation and subsequent reduction in the size of the region of interest. The mapping of this region to CDC Redberry (v2.0) gives insight into resistance genes present in the region. A total of 21 genes, comprised of seven zinc finger‐containing, six PPR‐containing, three transmembrane, three TIR‐domain‐containing, and two protein kinases, were annotated in this region. Only three, a TIR‐NBS‐LRR homologue and the two LRR‐containing kinases, are classical RGAs. TIR‐NBS‐LRR are a subset of NBS‐LRR genes containing a Toll/interleukin‐1 receptor, a signaling component involved with protein‐protein interactions. NBS‐LRR genes are well known as resistance genes in gene‐for‐gene interactions, recognizing pathogen avirulence effectors and in turn triggering a defense response, typically intracellular but occasionally localized to the plasma membrane (McHale et al., 2006; Zhu et al., 2025). LRR‐containing kinases are a large family more broadly involved in recognition and signaling, both in plant development and stress response, including biotic stresses like pathogen recognition. In comparison to TIR‐NBS‐LRR, LRR kinases are usually found in the membrane, where they recognize and signal the presence of extracellular effectors (Diévart & Clark, 2004; He et al., 2018). Though plant defense is, as noted, not the only function members of this gene family play, LRR‐containing kinase involvement in pathogen recognition and defense is extremely well documented (Tang et al., 2017).

Besides qAB_2.1, seedling assay screens identified four QTL associated with AB resistance, and field screening identified three. All of these were specific to the isolates used. Only two of these shared potential similarity in location with previous studies, namely, qAB_3.1 and qAB_3.2. Using BLAST, the markers closely associated with loci AB_IH2.1 and AB_IH2.2 reported by Sudheesh et al. (2016) mapped across chromosome 3 on reference genome CDC Redberry (v2.0), AB_IH2.2 between 86.4 and 92.9 Mbp, and AB_IH2.1 from a low of 254.0 Mbp to a high of 428.2 Mbp. Thus, AB_IH2.1 is collocated with QTL qAB_3.1, whereas AB_IH2.2 is distally located from qAB_3.2 on the CDC Redberry genome. QTL qAB_3.1 and qAB_3.2 are distinct from one another, and qAB_3.2 is in response to Pathotype 2 inoculation, whereas qAB_3.1, AB_IH2.1, and AB_IH2.2 were observed in response to Pathotype 1 inoculation. As such, understanding the conditions that the gene underlying this QTL functions under, and whether it is analogous to either of the AB_IH2 genes, would be beneficial to its usage in future resistance breeding.

No other QTLs were associated with markers from previous studies. Curiously, outside of qAB_3.2, all Pathotype 2 resistance QTLs were associated with ILL6002. This could be interpreted as instances where Indianhead carries susceptibility elements, though for moderate and minor QTL like these, the distinction generally lies in how common they are to a broader population. As ILL6002 is highly and consistently susceptible, and by the frequency distribution presented in Figure 1, we speculate Indianhead to be the more plausible origin of overall resistance, although further study would be required to determine the more appropriate label. It is also possible that ILL6002 carries other traits not directly linked to AB resistance, but which benefit the overall health of the plant enough to aid in withstanding infection (such as environmental adaptation), or that there are resistance elements here that only effectively function in the context of an Indianhead background. As ILL6002 carries agronomically favorable traits despite its poor AB resistance, and as Indianhead is used primarily for AB resistance, markers to select for or against these loci would be valuable in breeding.

The variation in the many QTL present and absent between experiments could result from many different factors. It is of some interest that, despite the complete lack of shared QTL between Pathotype 2 isolates *Al*Kewell and WAE21002‐1, Spearman's rank correlation testing found a weak but statistically significant positive correlation between the phenotypic scores of both isolates. This may indicate the presence of additional loci benefiting disease resistance against these isolates, which do not meet the statistical threshold with current results, the source or sources of which might be clarified through additional phenotypic screening. For the QTL that have been found here and which differ between experiments performed under the same conditions, the isolate, and possibly the pathotype, is the most likely explanation. Between field and seedling assays, these factors include individual isolates, age or growth stage of the plant, differing environmental conditions such as soil, water, and weather, and interacting organisms, such as competition from weed species, animal predation, wild diseases, and soil microbiota. Although low levels of Sclerotinia disease symptoms were suspected under field conditions, scoring of these symptoms produced no significant QTL. Plant‐microbial interactions, including instances between legumes and commercial rhizobial inoculant, have previously been observed to be capable of inducing plant‐wide immune response prior to pathogen infection, effectively “priming” the defensive pathways associated with pathogen response in one of two ways (Ranjbar Sistani et al., 2017). Induced systemic resistance is associated with JA and ethylene defensive signaling, and systemic acquired resistance with salicylic acid signaling, both of which have been implicated previously in lentil defense to *A. lentis* infection (Khorramdelezad, 2019; Ranjbar Sistani et al., 2017; Sari et al., 2018).

In addition to the AB resistance QTL, opportunistic screening of a powdery mildew infection identified a single QTL on LG 3, corresponding to an analogous region on chromosome 3. Resistance to infection was associated with ILL6002 genotype in the region of interest, or potentially, susceptibility was associated with Indianhead. Future investigation into the efficacy of this resistance may validate functional use or allow avoidance if it is a point of susceptibility. A weak but significant negative correlation was observed between powdery mildew infection and isolate WAE21002‐1, likely reflecting genetic linkage expected in the case of two QTL in close proximity, namely, qPM_3.1 and qAB_3.2. The infection analyzed in this study is an occasional but recurring problem in the glasshouse facility in which it was observed. It is believed to have originated from field pea (*Pisum sativum*), as the issue first and only emerged after an extensive infection within a field pea population. The species was unable to be confirmed, but all four *Erysiphe* species capable of infecting field pea are similarly capable of infecting lentil, and all have been recorded previously in Australia. The validity of *E. diffusa* has been questioned on the basis of a purely morphological identification but has not been further investigated (Attanayake, 2008; Banniza et al., 2004). *Erysiphe diffusa* has not yet been recorded in Western Australia, and *E. polygoni* and *E. trifolii* only on other legumes, specifically on clover (*Trifolium* sp.) and lupin (*Lupinus* sp.) for the former and on hairy vetch (*Vicia villosa* Roth) and *Sesbania* sp. for the latter (Cunnington et al., 2004; Shipton, 1967; Thomas & Coutts, 2018). As infections by *E. pisi* have been diagnosed on multiple legume hosts in Western Australia, including field pea (*Pisum sativum* L.) (GRDC, 2018), this is the most likely contributor to the case reported here. However, in the event that qPM_3.1 draws interest for practical use, confirming the species would be advisable.

Few historical studies on powdery mildew resistance in lentil are readily available to aid modern breeders and researchers. Current knowledge of powdery mildew resistance in lentil is comprised of two small collections of resistant lines and the locations of mildew resistance locus O (MLO) genes homologous to those in *Medicago truncatula* (Polanco et al., 2018). The first notable set of known resistant lines originates from the work of the Regional Pulse Improvement Project in Iran, while the second includes wild *Lens* accessions from taxa *Lens orientalis*, *Lens ervoides*, and *Lens nigricans* (Genesys, 2023; Gupta & Sharma, 2006; Khare, 1981; Mishra, 1973). Fifteen homologues of *Medicago truncatula* MLO genes have been identified across the lentil genome (Polanco et al., 2018). Their location in the CDC Redberry (v2.0) genome assembly is shown in Figure S7, along with the location of qPM_3.1 in this study. MLO genes are widely conserved in plants, generally containing one calmodulin‐binding domain and seven transmembrane domains (Kusch et al., 2016). Mutation of these genes is frequently associated with gain or loss of resistance to powdery mildew, but the location of the QTL identified in this study does not correlate with the location of any of the 15 MLO genes previously described (Kusch & Panstruga, 2017; Pavan et al., 2011; Polanco et al., 2018; Yundaeng et al., 2020). While the qPM_3.1 region in the lentil reference genome does not harbor any MLO genes, the homologous region in the Indianhead or ILL6002 genome feasibly could. Alternatively, the cause may be a different gene, or genes, entirely, as while MLO genes are the most frequently involved genes with mildew resistance and susceptibility, they are not involved exclusively (Bhosle & Makandar, 2021; Waengwan et al., 2024).

## 5 CONCLUSION

To conclude, the AB resistance in Indianhead was found to be effective against Pathotype 1 isolates in both controlled environment seedling disease assays and mature‐plant field disease assays. The major QTL for Pathotype 1 resistance is located on chromosome 2, and along with the two minor loci on chromosomes 3 and 5, explains 80.5% of the resistance phenotype. The markers flanking and under the identified QTLs are part of the pulse SNP chip and thus can be utilized directly and at immediate notice by the lentil breeding programs to trace these loci in the breeding program pedigree. Sequencing the Indianhead and ILL6002 genomes would also allow greater insight into the identification of AB and PM resistance gene candidates, and the development of perfect markers to implement on the SNP chip for the future breeding of enhanced disease resistance in lentil.

## AUTHOR CONTRIBUTIONS

**Em L. Thackwray**: Data curation; formal analysis; investigation; methodology; resources; validation; visualization; writing—original draft. **Bernadette M. Henares**: Conceptualization; data curation; investigation; methodology; project administration; supervision; writing—review and editing. **Christina R. Grime**: Data curation; investigation; methodology. **Bethany L. Clark**: Formal analysis; investigation; methodology. **Robert C. Lee**: Conceptualization; data curation; formal analysis; investigation; methodology; supervision; writing—review and editing. **Lars G. Kamphuis**: Conceptualization; data curation; funding acquisition; investigation; project administration; supervision; writing—review and editing.

## CONFLICT OF INTEREST STATEMENT

The authors declare no conflicts of interest.

## Supporting information




**Figure S1** Seed coat (testa) colouration from both parental accessions and F_2_ hybrid material, where F_2_ colour and pattern is determined by the F_1_ parent. Testa colour was used as ILL6002 and Indianhead cotyledon is the same colour (yellow).

**Figure S2** A simplified illustration of the environments used for each generation in progressing hybrid seed from F_1_ to F_6_.

**Figure S3** Marker distribution on seven linkage groups that correspond to the seven chromosomes of lentil of the genetic map of the ILL6002 × Indianhead recombinant inbred line (RIL) population.

**Figure S4** A heatmap demonstrating recombination and linkage between markers in the constructed linkage groups of the ILL6002 × Indianhead recombinant inbred line population. The heat map reflects the recombination relationship between markers in each linkage group. Each cell represents the recombination rate of two markers. Blue colour indicates a lower recombination rate while red colour indicates higher recombination rate and gradient in between as indicated.

**Figure S5** Comparison of the marker order as placed on the seven constructed linkage groups of the ILL6002 × Indianhead linkage map (left) and the seven chromosomes of the physical map the markers were developed on, CDC Redberry (v2.0) (right).

**Figure S6** Boxplots demonstrating phenotypic variance of z‐standardised values collected, grouped by the homozygous parental allelic variant of the respective marker located at the peak of each QTL. The isolate used is indicated on the Y axis of each plot, with isolates P94‐24 and AlKewell scored on seedlings under controlled environment conditions, and isolates WAC12393‐1 and WAE21002‐1 scored on mature plants under field conditions. Larger scores indicate greater disease.

**Figure S7** Distribution of Lens culinaris Mildew Locus O (MLO) genes (purple) as identified in Polanco et al. (2018) on the CDC Redberry (v2.0) lentil genome, and the peak of QTL qPM_3.1 (green) as identified in this study.

**Table S1** Genotypic data used in analysis. “RR” indicates alleles received from the resistant parent, and “SS” alles received from the susceptible parent.

**Table S2** Quality control procedures conducted on the markers received from the 30K Pulse SNP array, including the total number of markers removed at each step, and the remaining markers following removal.
**Table S3** Results of Spearman's Rank Correlation testing to compare phenotypic values collected between isolates. The Rho statistic, given in the bottom left half of the table, is indicative of the magnitude of the correlation, while the corresponding p‐value, given in the upper right half of the table, is indicative of whether this correlation is significant, where p < 0.05. The magnitude of the Rho statistic is generally considered as follows: 0.00‐0.19 = “very weak”, 0.20‐0.39 = “weak”, 0.40‐0.59 = “moderate”, 0.60‐0.79 = “strong”, 0.80‐1.00 = “very strong”, where a positive number is indicative of a positive correlation, and a negative number is indicative of a negative correlation.
**Table S4** Phenotypic data used in analysis

**Supplementary Data 1** A spreadsheet, each page containing the genes annotated on the CDC Redberry (v2.0) genome assembly, within each region of interest. Cells filled in blue denote plant defence‐associated genes, and those with orange text indicate classical resistance gene analogues. The name of each page indicates the respective QTL, and the associated *A. lentis* isolate, or powdery mildew where appropriate.

## Data Availability

All data generated or analyzed during this study are included in this published article and its Supporting Information files.
