# Supplementary material for: Genetic mapping of Ascochyta blight resistance in an ILL6002 × Indianhead lentil mapping population
Source: Plant Genome. 2025 Aug 30;18(3):e70097. doi: 10.1002/tpg2.70097 (PMC12397902; doi:10.1002/tpg2.70097)
Supplement: Supplementary file 1 [file TPG2-18-e70097-s005.pdf]

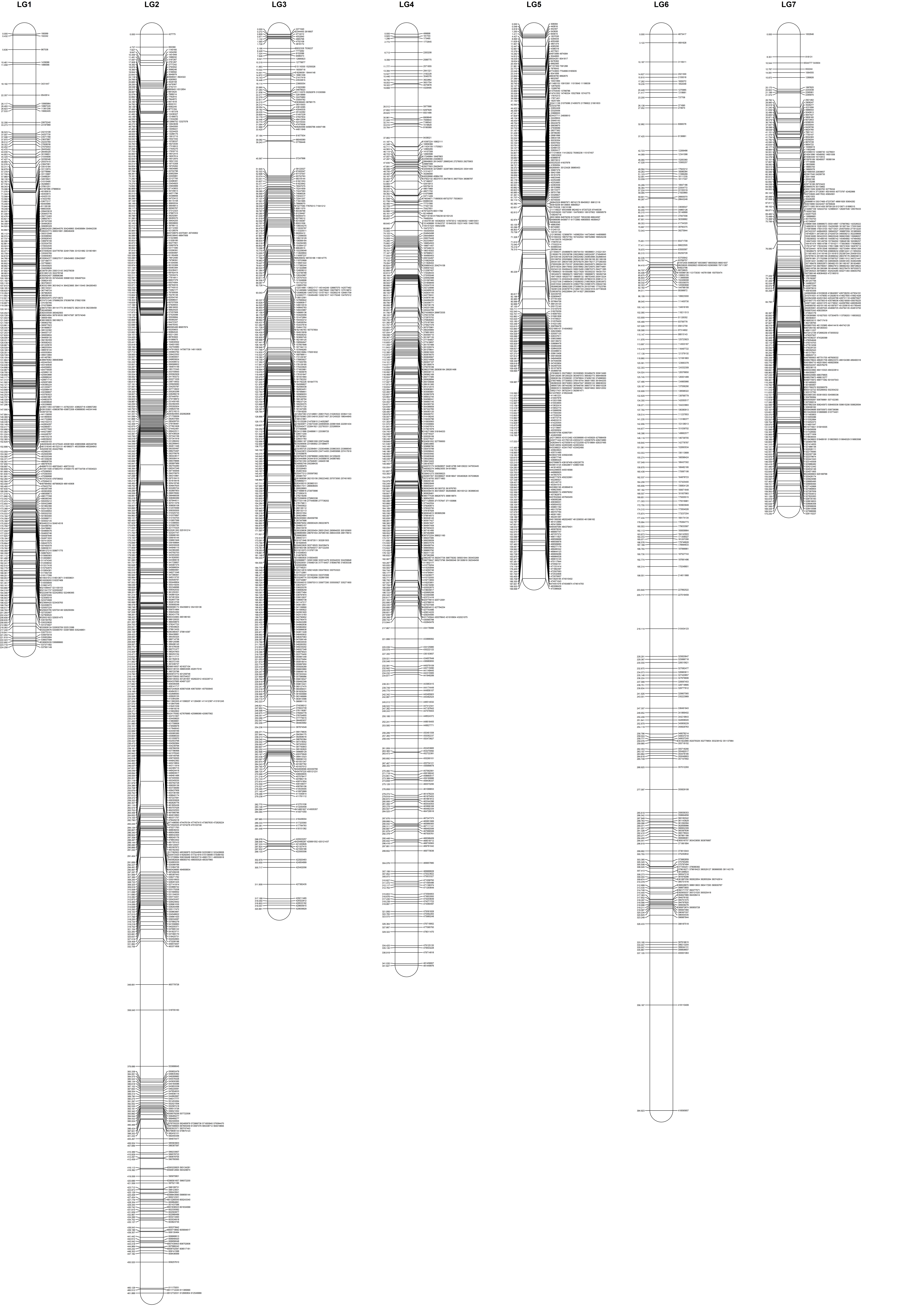
LG1
LG2
LG3
LG4
LG5
LG6
LG7
