# Supplementary figures and images for: Genetic mapping of Ascochyta blight resistance in an ILL6002 × Indianhead lentil mapping population
Source: Plant Genome. 2025 Aug 30;18(3):e70097. doi: 10.1002/tpg2.70097 (PMC12397902; doi:10.1002/tpg2.70097)

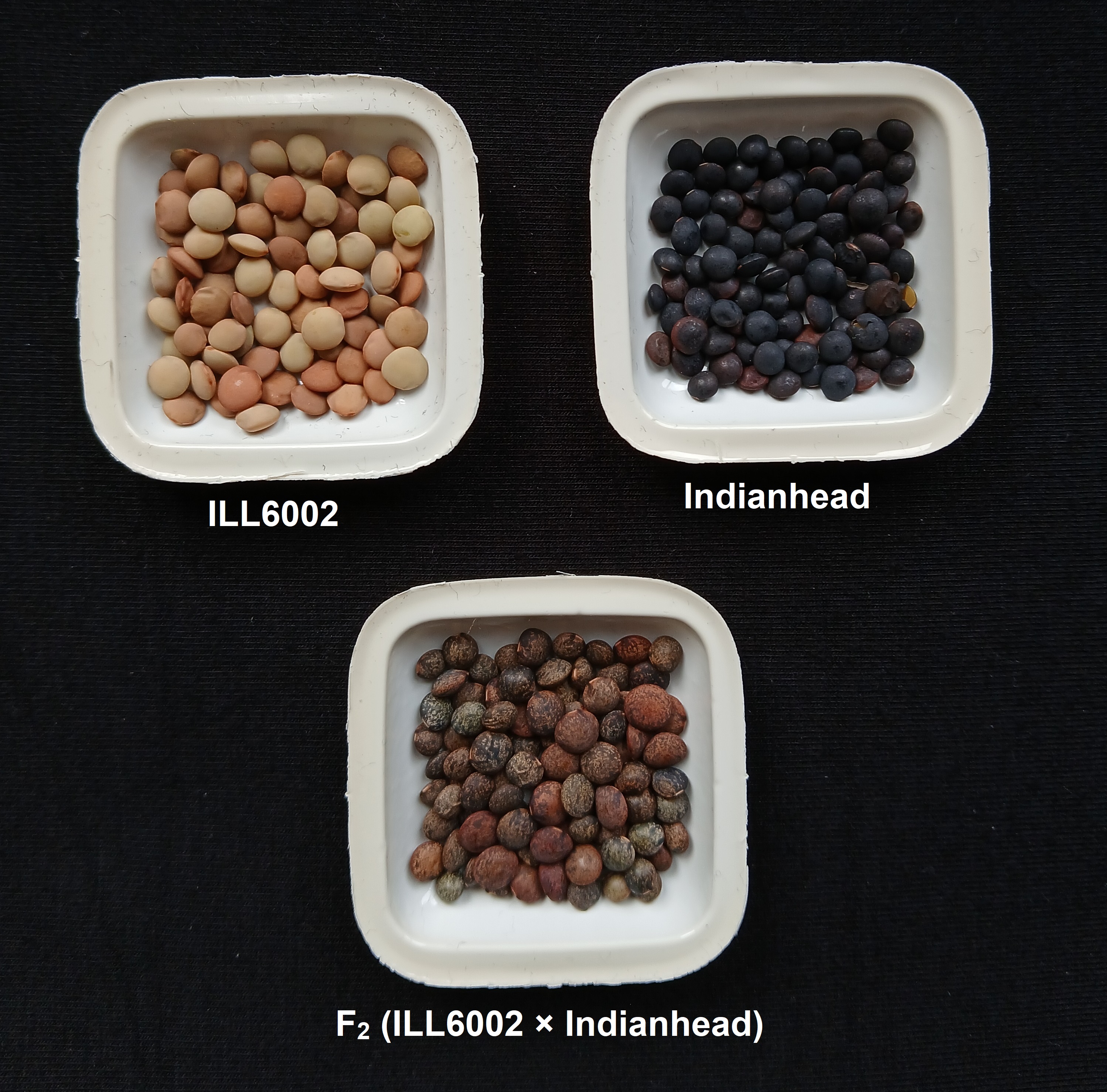

Supplement: Supplementary file 1 — Figure S1 Seed coat (testa) colouration from both parental accessions and F2 hybrid material, where F2 colour and pattern is determined by the F1 parent. Testa colour was used as ILL6002 and Indianhead cotyledon is the same colour (yellow). [file TPG2-18-e70097-s010.jpg]

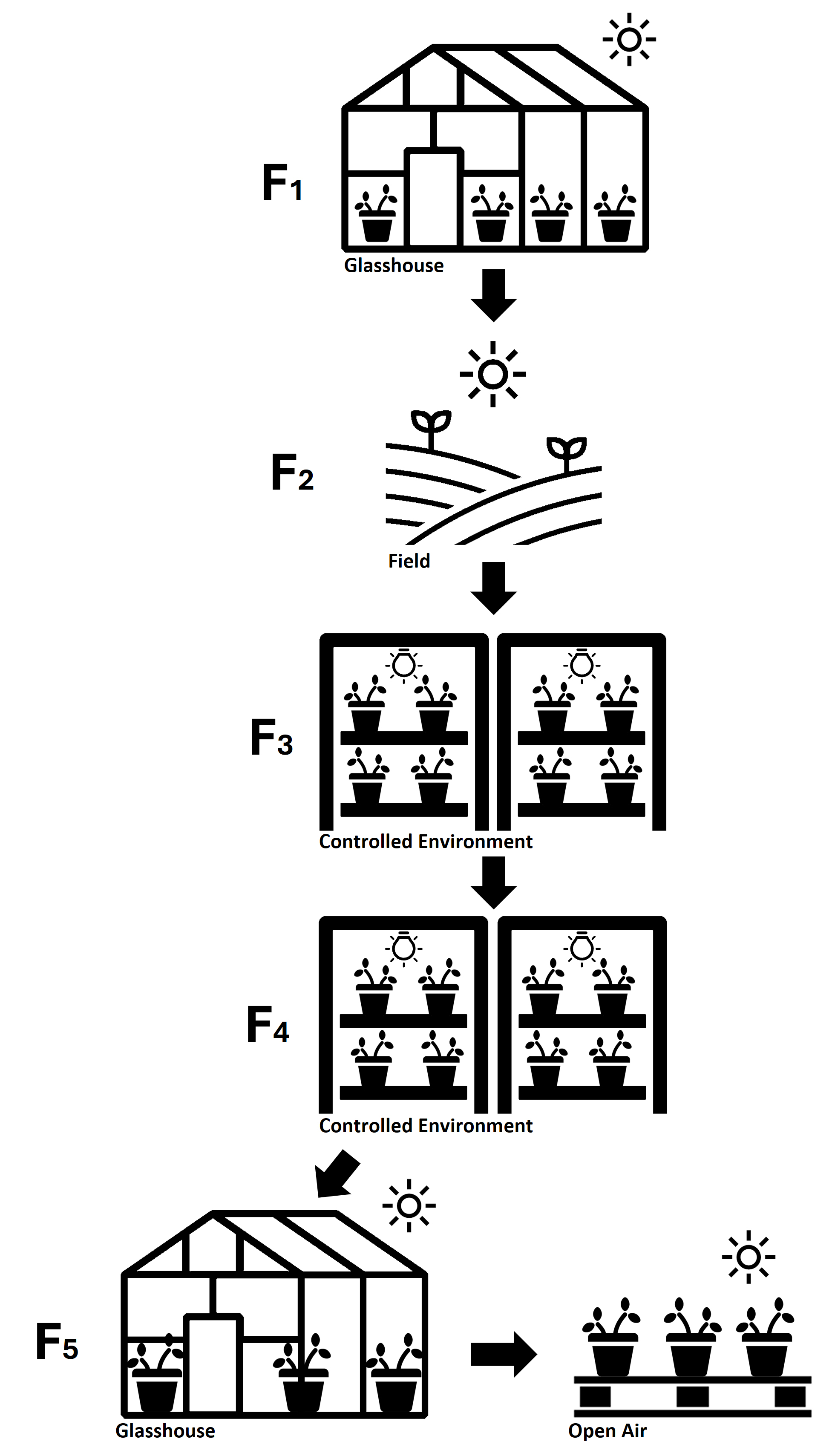

Supplement: Supplementary file 2 — Figure S2 A simplified illustration of the environments used for each generation in progressing hybrid seed from F1 to F6. [file TPG2-18-e70097-s002.jpg]

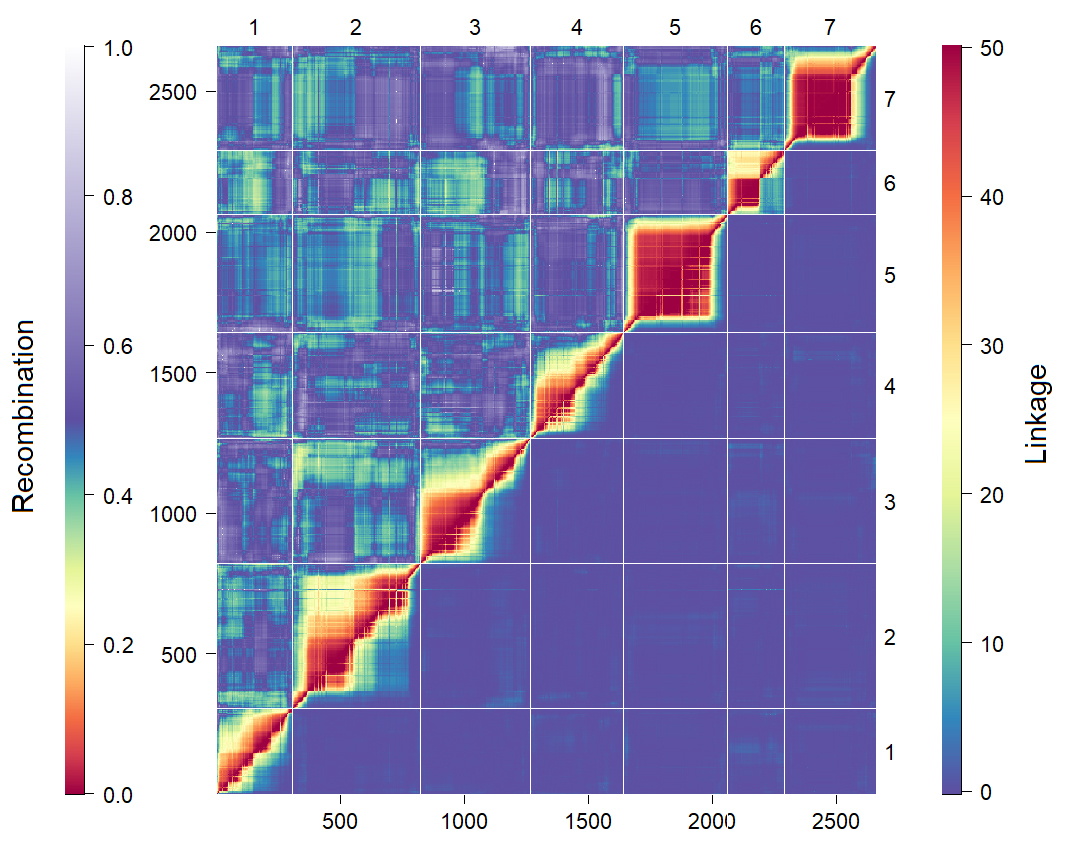

Supplement: Supplementary file 4 — Figure S4 A heatmap demonstrating recombination and linkage between markers in the constructed linkage groups of the ILL6002 × Indianhead recombinant inbred line population. The heat map reflects the recombination relationship between markers in each linkage group. Each cell represents the recombination rate of two markers. Blue colour indicates a lower recombination rate while red colour indicates higher recombination rate and gradient in between as indicated. [file TPG2-18-e70097-s008.jpg]

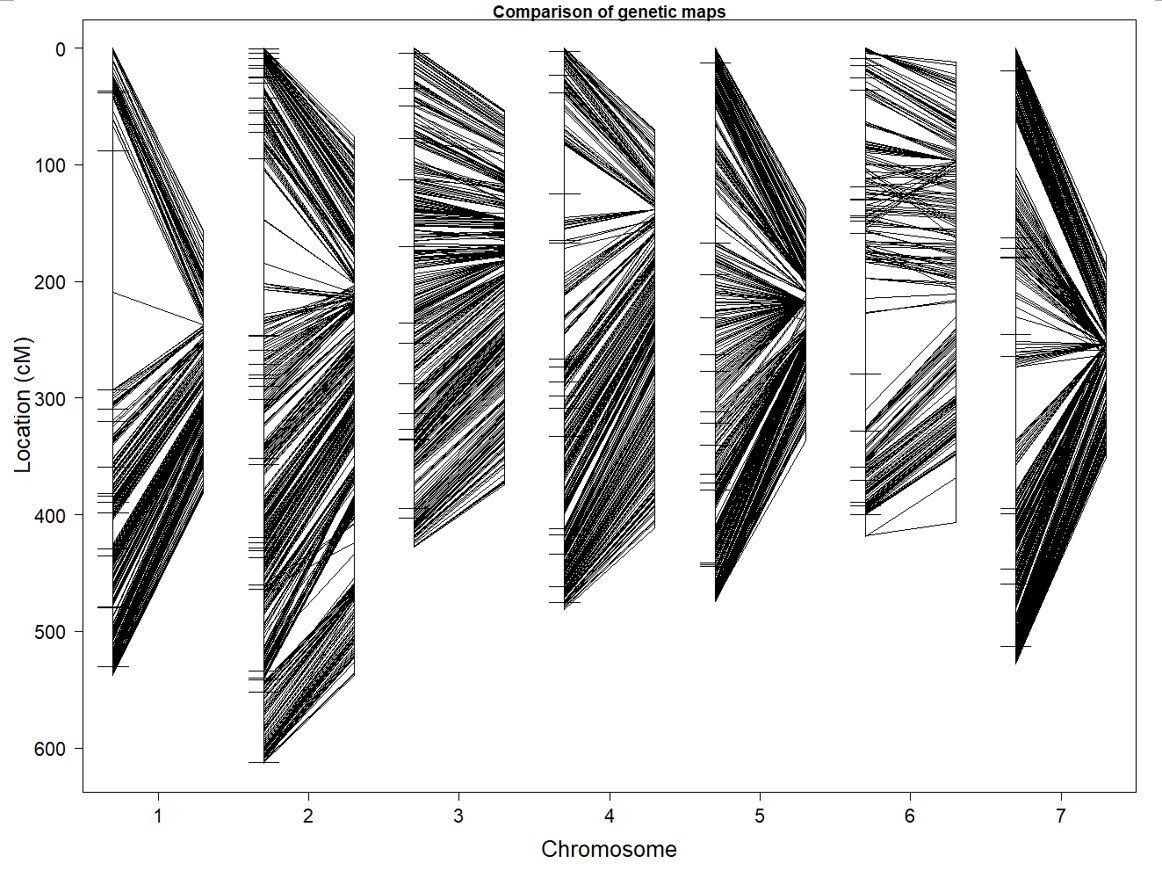

Supplement: Supplementary file 5 — Figure S5 Comparison of the marker order as placed on the seven constructed linkage groups of the ILL6002 × Indianhead linkage map (left) and the seven chromosomes of the physical map the markers were developed on, CDC Redberry (v2.0) (right). [file TPG2-18-e70097-s007.jpg]

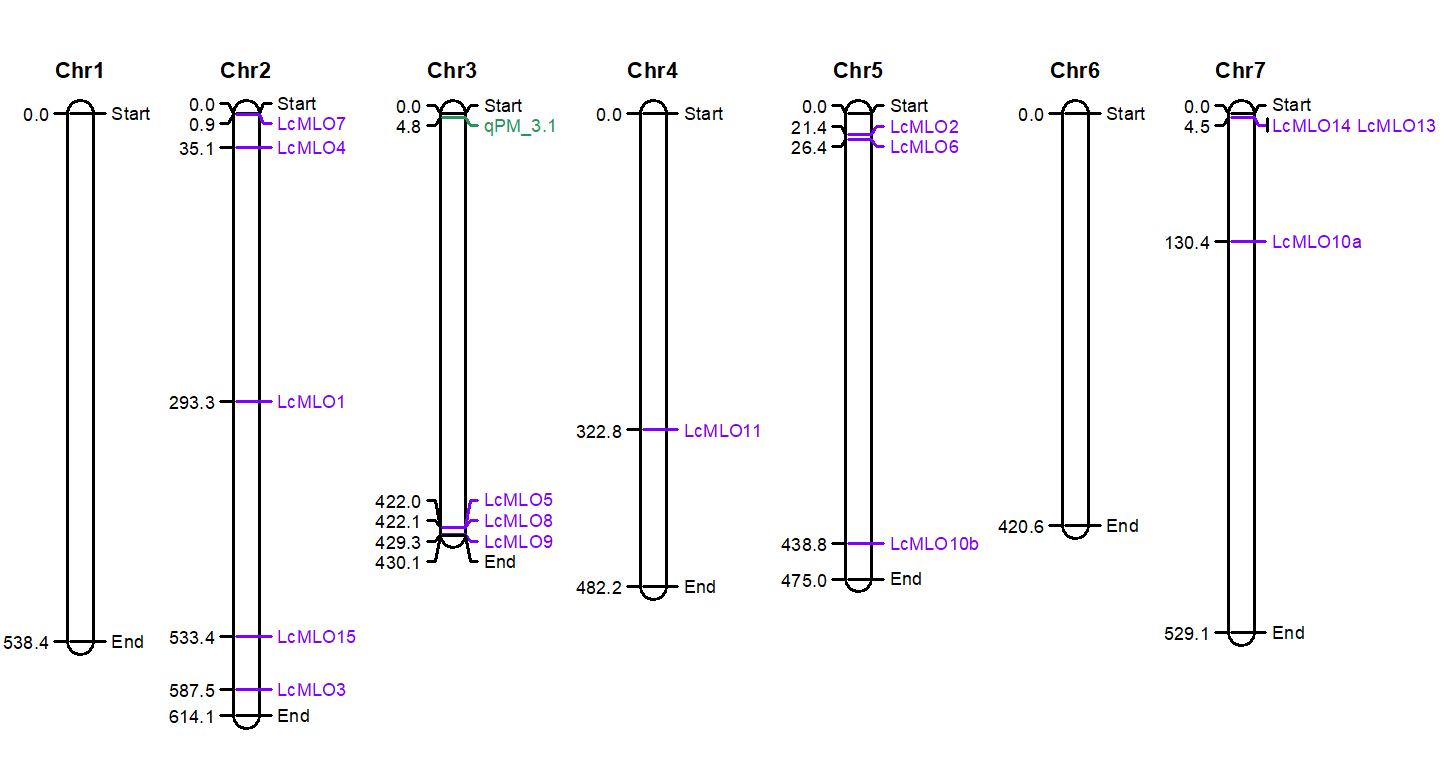

Supplement: Supplementary file 7 — Figure S7 Distribution of Lens culinaris Mildew Locus O (MLO) genes (purple) as identified in Polanco et al. (2018) on the CDC Redberry (v2.0) lentil genome, and the peak of QTL qPM_3.1 (green) as identified in this study. [file TPG2-18-e70097-s004.jpg]
